# Construction and optimization of a genomic selection model for total sugar content in tobacco

**DOI:** 10.3389/fgene.2026.1886099

**Published:** 2026-07-23

**Authors:** Yue Yang, Qiang Xu, Jincun Fu, Linjie Guo, Zexiang Huang, Huan Si, Hao Wang, Guanwang Shen, Yanjun Zan, Zongyu Hu

**Affiliations:** 1 China Tobacco Jiangsu Industrial Co., Ltd., Nanjing, China; 2 Tobacco Research Institute, Chinese Academy of Agricultural Sciences, Qingdao, China; 3 College of Agronomy Qingdao Agricultural University, Qingdao, China; 4 Shandong Tobacco Corporation, Jinan, Shandong, China; 5 Integrative Science Center of Germplasm Creation in Western China (Chongqing), Science City, Biological Science Research Center, Southwest University, Chongqing, China; 6 Department of Plant Physiology, Umeå Plant Science Center and Integrated Science Lab, Umeå University, Umeå, Sweden

**Keywords:** genome-wide association analysis, genomic selection, machine learning, tobacco, total sugar

## Abstract

Total sugar is one of the chemical indicators related to tobacco quality. However, due to its composition of various oligosaccharides, disaccharides, and polysaccharides, total sugar lacks a clear genetic target, making conventional breeding approaches for its improvement particularly challenging. Therefore, conducting genomic selection (GS) studies on total sugar content holds significant importance for the development of high-sugar tobacco varieties. In this study, 2,604 tobacco germplasm accessions with broad genetic diversity, sourced from the National tobacco Germplasm Repository, were used as experimental materials. High-throughput sequencing technologies were employed to perform comprehensive genetic evaluation and construct genomic selection models for total sugar content. Sixteen mainstream genomic prediction models were assessed through five-fold cross-validation to develop a high-accuracy prediction framework. Among these models, the Gradient Boosting Machine (GBM) achieved the highest prediction accuracy for total sugar content (0.85), followed by the rrBLUP model (0.81). Comparative analysis of computational speed and resource consumption revealed that GBM maintained rapid computation and low resource usage even in large sample sizes, demonstrating strong stability and superior performance. Considering all factors, GBM was preliminarily identified as the optimal model for predicting total sugar content in tobacco. The application of high-accuracy genomic prediction is expected to overcome the challenges of phenotypic evaluation in breeding programs and significantly enhance the efficiency of selecting high-sugar tobacco varieties.

## Introduction

Tobacco (*Nicotiana tabacum L*.) is an important economic crop in China and is widely cultivated across both northern and southern provinces. At present, tobacco is grown and produced in 24 provinces and autonomous regions, forming six major tobacco-producing areas: North China, Northeast China, East China, Central and South China, Southwest China, and Northwest China (Yu et al., 2026). Among these regions, Yunnan is the largest tobacco-producing area in China. Tobacco is also a major component of Yunnan’s agricultural industry and plays an important role in the province’s economic development. In 2019, the total tobacco leaf production in Yunnan reached 835,400 tons, accounting for 38.79% of the national total tobacco production of 2,153,400 tons. Despite its high economic value, with the continuous increase in consumer demand for tobacco quality, the genetic improvement of total sugar content has gradually become an important focus in tobacco breeding (Tong et al., 2021; Ullah et al., 2025; Tsaliki et al., 2023).

Total sugar is one of the chemical traits associated with tobacco quality and has significant effects on sensory characteristics such as combustibility, smoking taste, aroma, and smoke softness (Tong et al., 2020; Kumar et al., 2024). An appropriate total sugar content helps improve flavor balance and smoking quality. In addition, during cigarette processing, the pyrolysis products of sugar compounds can participate in the formation of aroma-related substances, thereby enhancing overall product quality. Therefore, achieving proper regulation of sugar content has become one of the key targets in tobacco breeding. However, total sugar represents the sum of various oligosaccharides, disaccharides, and polysaccharides. Its metabolism is regulated by a complex network, and the target substances involved remain unclear. In addition, the genetic architecture of total sugar is complex, and major-effect loci have not been clearly identified. As a result, conventional phenotypic selection and marker-assisted selection (MAS) face clear limitations in the genetic improvement of total sugar (Collard and Mackill, 2008). Therefore, more efficient and systematic breeding strategies are needed to advance genetic improvement for this trait.

Genomic selection (GS), also referred to as genomic prediction (GP), is an extension of marker-assisted selection to the whole-genome level (Meuwissen et al., 2001; Montesinos-López and Kismiantini and Montesinos-López, 2023). It mainly uses a large number of genome-wide molecular markers together with phenotypic data from a reference population to build prediction models, estimate the breeding value contributed by markers across the genome, and then predict the breeding values of offspring individuals using marker data alone for selection (Chao et al., 2025; Ma et al., 2025). Implementation of GS requires two key populations: a training population (TP) and a breeding population (BP), both genotypic and phenotypic data are collected to train a regression model that estimates the effects of genetic markers across the whole genome. In contrast, each sample in the BP only needs to be genotyped, and its breeding value for the target trait can then be predicted using the trained model (Alemu et al., 2024). Individuals in the BP are subsequently ranked according to their estimated breeding values, and those with superior values are selected. GS was first applied in animal breeding and was later introduced into crop breeding, including major crops such as maize (Yan et al., 2021a; He et al., 2025), rice (Ma et al., 2024; Wang et al., 2025a) and wheat, where it has greatly improved the efficiency of genetic improvement. Taken together, GS enables accurate prediction of total sugar content using genotypic data without the need for phenotypic evaluation, and therefore provides an important approach for breeding tobacco varieties with high sugar content.

In recent years, genomic selection has gradually been introduced into tobacco breeding, although its application remains largely exploratory. Previous studies have shown that tobacco quality-related traits, including total sugar, are controlled by a complex genetic architecture. For example, Zhang et al. identified SNP markers associated with several leaf chemical traits, including total sugar, based on a GWAS analysis of diverse tobacco germplasm, providing evidence for the genetic basis of these traits (Tong et al., 2020). Tong et al. further analyzed QTL distributions for multiple agronomic and chemical traits using a high-density genetic map and, for the first time, evaluated the performance of several GS models in tobacco (Tong et al., 2021). Their results supported the feasibility of GS for tobacco genetic improvement and highlighted its potential value in molecular breeding.

In this study, 2,604 tobacco germplasm accessions with broad genetic diversity were used to conduct a systematic genetic evaluation of total sugar content. The prediction accuracy of 16 genomic selection models for total sugar was assessed using five-fold cross-validation, and a genomic selection model for total sugar in tobacco was established. This study provides a theoretical and methodological basis for improving total sugar through GS.

## Materials and methods

### Experimental materials and field design

A total of 2,604 tobacco germplasm accessions with broad genetic diversity were selected for this study, including backbone varieties approved or registered over the past 30 years, genetic materials with important breeding value from industry genome programs and major biological breeding projects, as well as widely cultivated varieties. A field experiment was conducted in 2022 in Longshan County, Hunan Province, China. The field trial was arranged in a randomized complete block design with two replicates. Each plot covered 22.0 m^2^, with a row spacing of 1.1 m, a plant spacing of 0.6 m, and a row length of 10.0 m. Two rows were planted in each plot, with one plant per hill and more than 20 plants per plot in total. Seeds were sown around February 27. When plants reached technological maturity in mid-September, leaves were harvested in three rounds. Field management, including irrigation and fertilization, followed local conventional cultivation practices.

### Phenotype identification

Middle leaves were harvested from each tobacco accession at the appropriate maturity stage. After harvest, the leaves were subjected to heat treatment and drying to inactivate enzymatic activity and obtain dry samples for chemical analysis, thereby minimizing the effects of postharvest physiological changes on sugar determination. The dried leaf samples were then ground and passed through a 40-mesh sieve to obtain uniform powder. Sample preparation conditions were strictly controlled to ensure comparability among samples. Total sugar content was determined at the Quality and Safety Research Center, Tobacco Research Institute, Chinese Academy of Agricultural Sciences, using the continuous flow method according to the Chinese tobacco industry standard YC/T 159–2019 for the determination of water-soluble sugars in tobacco and tobacco products. Three technical replicates were performed for each sample, and the mean value was used as the phenotypic value. Total sugar content was expressed as grams of sugar per 100 g of dry sample (g/100 g). Experimental conditions were strictly controlled throughout the analysis to ensure consistency among samples. The resulting phenotypic data were used for subsequent genetic analysis and genomic selection.

The narrow-sense heritability of total sugar content was estimated as:

$$h^{2}=\frac{\sigma_{A}^{2}}{\sigma_{A}^{2}+\sigma_{e}^{2}}$$

where $\sigma_{A}^{2}$ represents additive genetic variance, $\sigma_{e}^{2}$ represents residual variance.

### Genotype data processing

Genotyping of the 2,604 germplasm accessions was performed using genotyping-by-sequencing (GBS) (Elshire et al., 2011). Genomic DNA was first extracted using the CTAB method. After digestion with NlaIII and MseI, the resulting fragments were purified with AMPure XP beads and used for library construction. High-throughput sequencing was then carried out on the Illumina NovaSeq platform. Raw sequencing data were quality-filtered using fastp (Chen et al., 2018) to remove low-quality reads, and the clean reads were aligned to the ZY300 reference genome using BWA (Li and Durbin, 2009). Variants were identified based on SAMtools mpileup (Li et al., 2009). High-quality SNPs were initially screened using the following criteria: average sequencing depth >2, minor allele frequency (MAF) > 0.03, and missing rate <0.5 for both individuals and loci (Poland et al., 2012; Lu et al., 2013). To improve genotype completeness, missing genotypes were imputed using Beagle (Browning and Browning, 2016), resulting in a final set of 98,667 high-quality SNP markers. Validation with whole-genome resequencing data showed that the average SNP calling accuracy reached 0.96, indicating high reliability.

### Principal component analysis

PCA was conducted using the prcomp function with scaled genotype data. The first two principal components (PC1 and PC2) were retained for visualization and downstream clustering. K-means clustering was applied to the PC1 and PC2 scores. The optimal number of clusters (k) was determined using the within-cluster sum of squares (WSS) elbow method, implemented via factoextra R package (Kassambara and Mundt, 2016). Based on the inflection point observed in the WSS curve, k = 3 was selected as the optimal cluster number.

### GWAS analysis

GWAS for total sugar content was performed using a mixed linear model implemented in GEMMA. The model incorporated SNP effects as fixed effects and controlled for population structure and genetic relatedness using principal components and a genomic relationship matrix. The genome-wide significance threshold was determined by Bonferroni correction based on the number of filtered SNPs. Manhattan and QQ plots were generated to display association signals and assess the effectiveness of population structure correction.

### Construction of the genomic selection model

Based on the filtered set of 98,667 SNP markers, genomic selection analysis was performed for total sugar content in tobacco, using total sugar content as the phenotypic trait. A total of 16 widely used genomic selection (GS) models were applied to the filtered genotypic and phenotypic data for prediction analysis. These models can be broadly classified into three categories.

The first category included linear models, namely, ridge regression (RR), least absolute shrinkage and selection operator (LASSO) (Ranstam and Cook, 2018), and elastic net (EN) (Zou and Hastie, 2005). These models are particularly useful when the underlying genetic architecture is expected to be sparse, as they can identify relevant markers while excluding unimportant ones. The second category included mixed linear models. These comprised additive models such as genomic best linear unbiased prediction (GBLUP) (Clark and Van Der Werf, 2013) and ridge regression best linear unbiased prediction (rrBLUP) (Endelman, 2011), non-additive kernel-based models such as reproducing kernel Hilbert space (RKHS) (Berlinet and Thomas-Agnan, 2011) and multi-kernel reproducing kernel Hilbert space (MKRKHS) (Yu et al., 2024), and Bayesian additive models including Bayes A (BA), Bayes B (BB), Bayes C (BC) (Webb et al., 2010), Bayesian ridge regression (BRR) (Shi et al., 2016), and Bayesian LASSO (BL) (Park and Casella, 2008). These models are based on different assumptions about the genetic architecture of the trait. Some are more suitable for traits controlled mainly by many small-effect genes, some for traits influenced by major-effect genes, and others for traits jointly controlled by both major- and small-effect genes. The third category consisted of nonlinear machine learning models, including deep neural network genomic prediction (DNNGP) (Wang K. et al., 2023), gradient boosting machine (GBM) (Yan et al., 2021b), random forest (RF) (Rigatti, 2017), and support vector machine (SVM) (Suthaharan, 2016). A key feature of these models is their ability to capture complex non-additive effects, making them potentially effective for traits influenced by genetic interactions.

Together, these 16 models from the three categories covered the major statistical and machine learning methods commonly used in current GS studies. They were selected to account for different possible genetic architectures of total sugar content and to provide a comprehensive evaluation for establishing a genomic selection framework for this trait in tobacco (Abdollahi-Arpanahi et al., 2020; Heslot et al., 2012). To improve the reproducibility of this study, all models were evaluated under the same five-fold cross-validation framework, and prediction accuracy was measured using the Pearson correlation coefficient between predicted and observed phenotypic values (Zhou et al., 2021; Wang H. et al., 2023). For the linear and Bayesian models, default parameter settings were used. For the machine learning and deep learning models, a grid search strategy was used for hyperparameter optimization. The main software packages and hyperparameter ranges used for each model are provided in Supplementary Table S1.

### Cross-validation and model evaluation

To evaluate the predictive performance of the 16 models, five-fold cross-validation (k = 5) was used. The population was randomly divided into five equal subsets (Wang et al., 2025b; Wang et al., 2026). In each round, 20% of the individuals were randomly assigned to the TP, which contained genotypic data only, while the remaining 80% were used as the TP, which included both genotypic and phenotypic data. The GS models were trained on the TP and then used to predict the phenotypic values of individuals in the TP (Zhang et al., 2019). Model performance was evaluated using the Pearson correlation coefficient (r), calculated as the correlation between predicted breeding values and observed breeding values. In addition, differences among models in computational efficiency, including running time and computational resource use under different sample sizes, were compared to identify the optimal GS model.

### Computational efficiency

Computational efficiency and resource consumption are important factors affecting the application of genomic selection models in large-scale breeding. Therefore, this study used computer-based simulations to compare the computational speed and resource requirements of different GS models when handling large datasets. All simulations were performed on a laptop equipped with a 12th Gen Intel processor (12 cores, 2.50 GHz), 16 GB RAM, and a 64-bit Windows 11 operating system. All analyses were conducted in the R environment (version 4.4.1).

## Result

### Genetic diversity assessment of the 2,604 accessions in the training population

**FIGURE 1:**
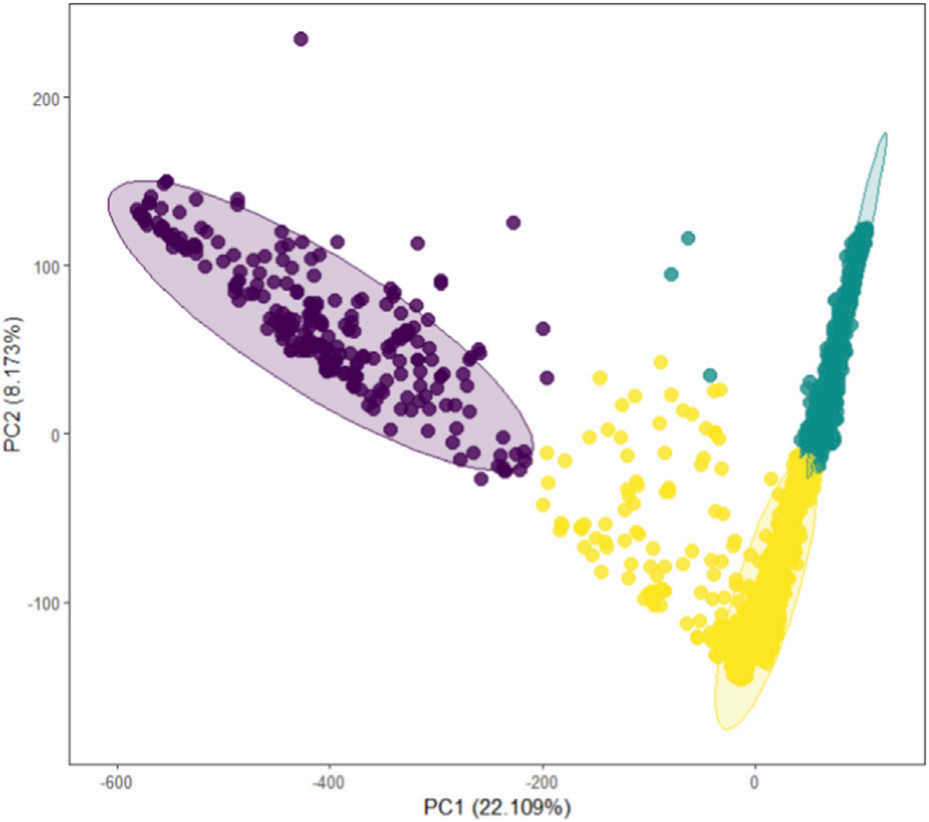
PCA analysis of total sugar traits in tobacco.

In this study, genotyping-by-sequencing was performed on 2,604 tobacco accessions from the National Tobacco Germplasm Bank. The materials included flue-cured tobacco (1,063 accessions), sun-cured tobacco (12 accessions), cigar tobacco (50 accessions), burley tobacco (133 accessions), oriental tobacco (68 accessions), air-cured tobacco (1,198 accessions), Maryland tobacco (11 accessions), and medicinal tobacco (69 accessions). Principal component analysis (PCA) (Patterson et al., 2006) showed that the population could be divided into three subgroups based on genetic similarity (Figure 1). The purple group consisted mainly of introduced tobacco varieties (294 accessions), the dark green group represented Chinese-bred varieties (1,106 accessions), and the yellow group included Chinese landraces and local varieties (1,204 accessions). These results indicate that the genetic structure of this population was mainly influenced by geographic origin and germplasm type, such as local varieties, bred varieties, and introduced varieties.

### Phenotypic variation and heritability of total sugar content

**FIGURE 2:**
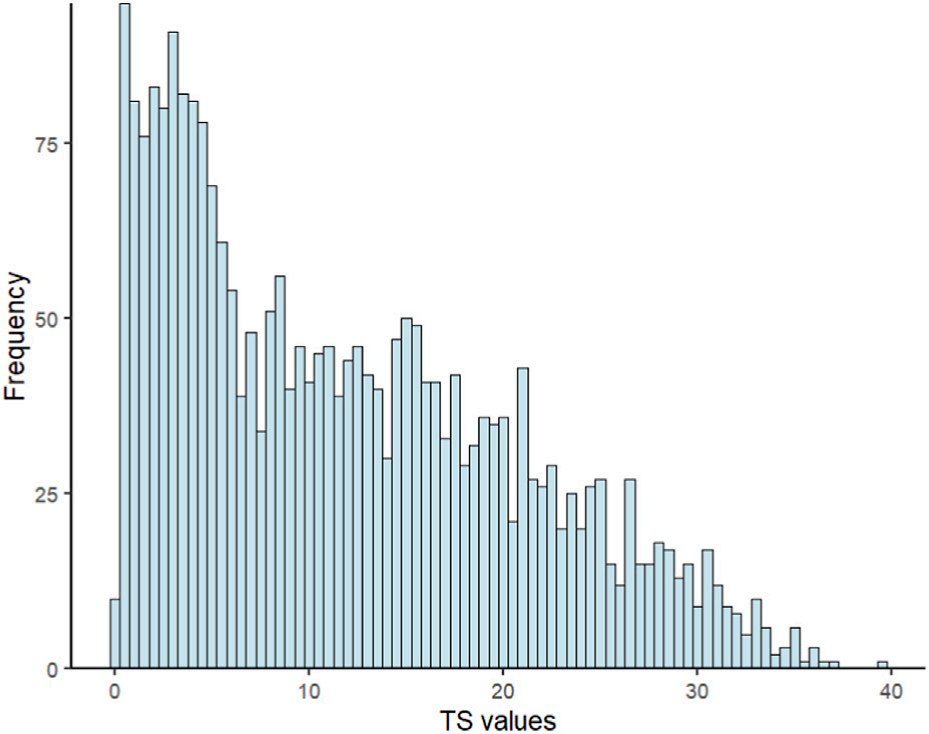
Phenotypic distribution and estimated heritability of tobacco total sugar content.

Phenotypic analysis of total sugar content in the 2,604 accessions showed that the values ranged from 0.07 to 39.68 mg/g, with a mean of 11.87 mg/g (Figure 2). Approximately 79% of the samples were distributed within the range of 0.07–20 mg/g. Among the major cultivated varieties in China, the total sugar contents of K326 and Yunyan 87 were 17.72 mg/g and 32.82 mg/g, respectively. These results indicate substantial phenotypic variation in total sugar content among Chinese tobacco varieties, introduced materials, and local germplasm resources. Heritability analysis showed that the narrow-sense heritability of total sugar content was h^2^ = 0.36 ± 0.03. The likelihood ratio test further indicated that the genetic effect was highly significant (LRT = 1,655.76, df = 1, P < 10^−100^) (Kang et al., 2008; Yang et al., 2011; Visscher et al., 2008). This suggests that total sugar content is a trait with moderate heritability, and that a considerable proportion of the phenotypic variation can be explained by genetic factors, indicating good potential for genetic improvement.

### GWAS reveals the polygenic architecture of total sugar content

**FIGURE 3:**
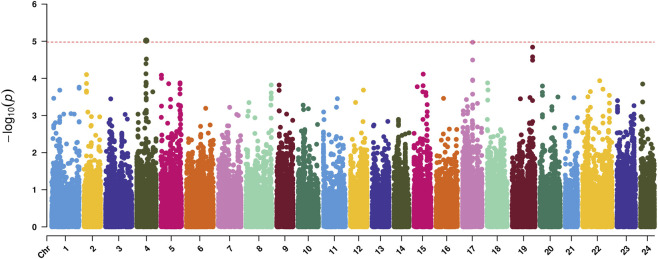
GWAS analysis of total sugar content in tobacco. The Manhattan plot shows SNP associations with total sugar content across the tobacco genome. The dashed line indicates the suggestive significance threshold of −log_10_(P) = 5. One significant signal was detected on chromosome 4 at 77, 398, 396 bp, while most signals remained below the threshold.

To further investigate the genetic basis of total sugar content in tobacco, a genome-wide association study (GWAS) was conducted using the phenotypic data from 2,604 accessions together with the genotypic data obtained by genotyping-by-sequencing. As shown in Figure 3, using a significance threshold of −log_10_ (P) ≥ 5, one significant association signal was detected at 77, 398, 396 bp on chromosome 4. However, this locus explained only a small proportion of the genetic variation, whereas most of the phenotypic variation appeared to be contributed by a large number of loci with smaller effects that did not reach genome-wide significance. Overall, these results suggest that total sugar content in tobacco has a polygenic genetic architecture characterized by many small-effect loci. In other words, this trait is likely regulated by the combined effects of multiple minor loci, possibly through additive effects and gene interactions, rather than being controlled by a single major-effect gene. This finding provides further evidence for understanding the genetic basis of total sugar content.

### Prediction performance of genomic selection models

**FIGURE 4:**
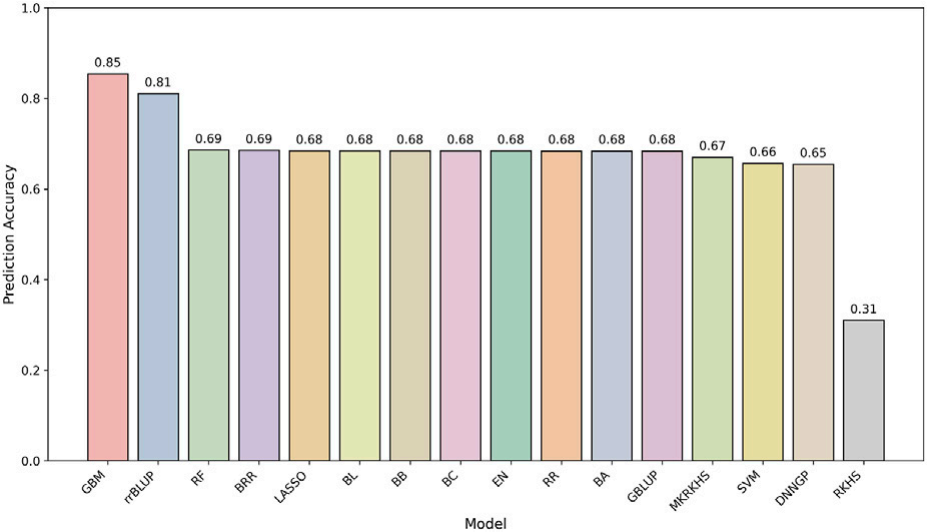
Prediction accuracy of the total sugar trait in tobacco evaluated using ten repetitions of five-fold cross-validation.

In this study, the predictive performance of 16 genomic selection (GS) models, including Bayes A, Bayes B, Bayes C, BL, BRR, GBLUP, rrBLUP, LASSO, GBM, EN, RR, RKHS, SVM, MKRKHS, RF, and DNNGP, was evaluated for total sugar content. Prediction accuracy was calculated as the average Pearson correlation coefficient across ten repeated five-fold cross-validations. As shown in Figure 4, except for RKHS, most models achieved prediction accuracies of around 0.65. Among them, the GBM model showed the best performance, with a prediction accuracy of 0.85, followed by the rrBLUP model, which reached 0.81. Overall, GBM was identified as the optimal model and can be considered the preferred approach for genomic prediction of total sugar content in tobacco.

### Computational efficiency of genomic selection models

**FIGURE 5:**
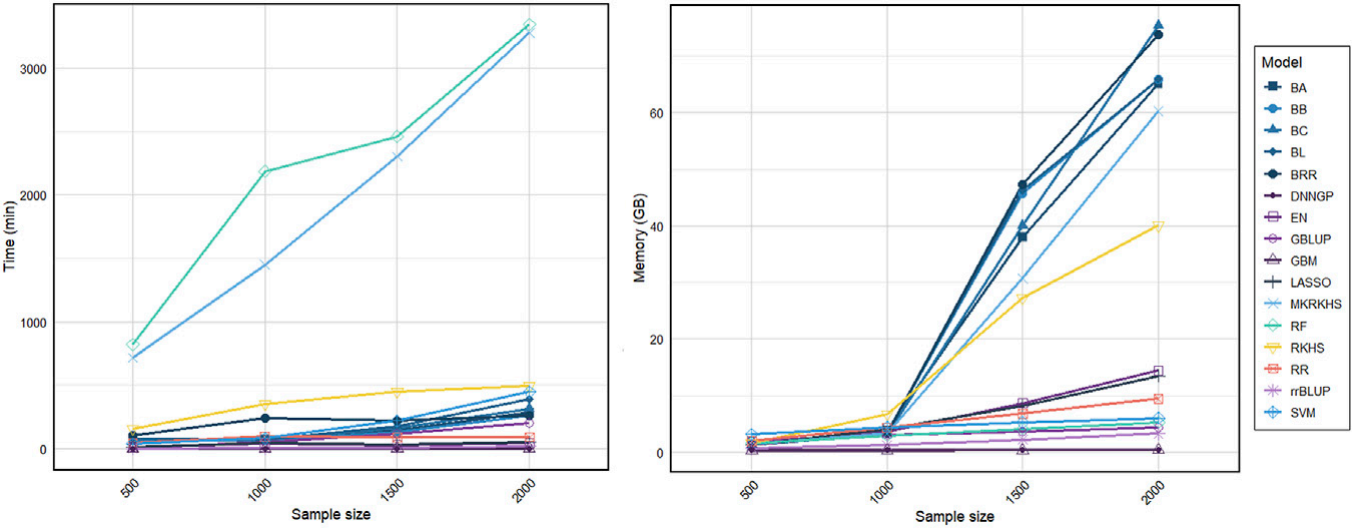
Comparison of operation speed and computing resources of different models.

Using random sampling, we generated multiple simulated populations with sample sizes of 500, 1,000, 1,500, and 2000. To ensure that the simulated genotypic data reflected the genetic structure of tobacco populations in real breeding programs, each simulated dataset was generated by random sampling with replacement from the 2,604 real tobacco accessions. All 16 models were applied to each simulated population, and five-fold cross-validation was used to evaluate running time (seconds), memory usage, and CPU utilization. As shown in Figure 5, the GBM model consistently showed high computational efficiency and low memory consumption across all sample sizes. In particular, when the sample size reached 2000, the GBM model used only 1.83 GB of memory.

### Field validation of the prediction model

**FIGURE 6:**
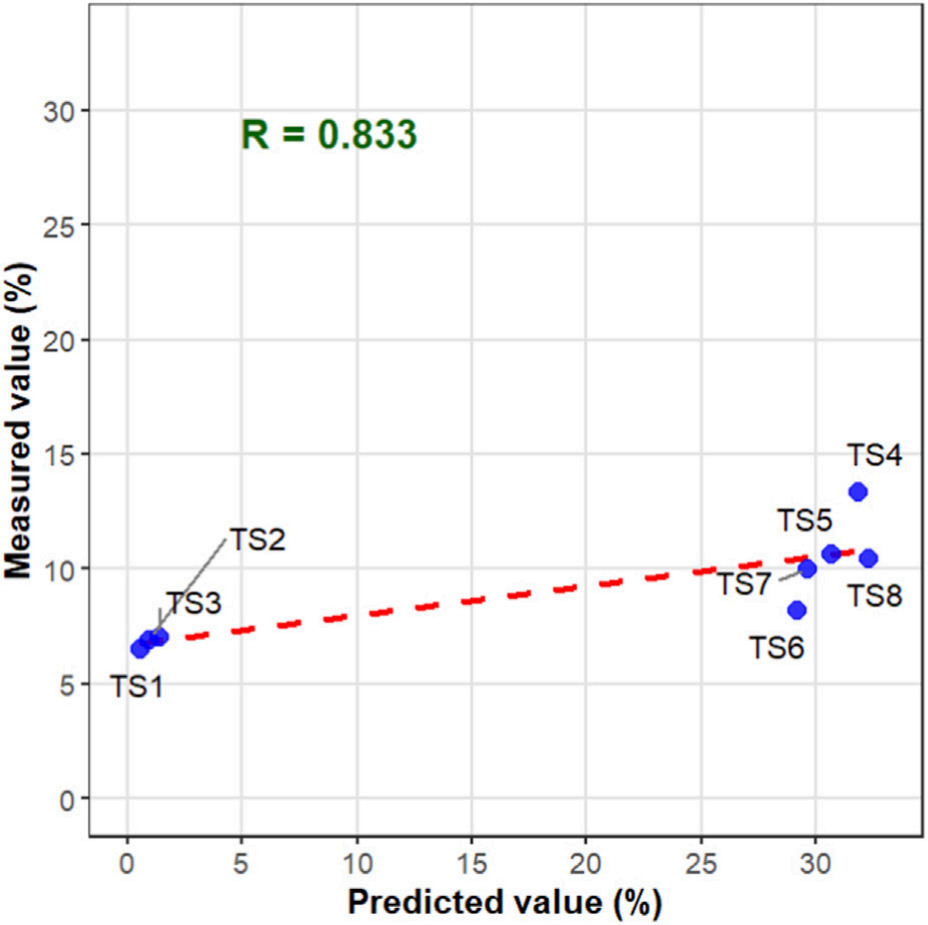
Prediction accuracy of total sugar content using GBM model.

To validate the prediction model for total sugar content in tobacco leaves, the GBM model was used to predict total sugar content for 5,500 accessions from the Chinese Tobacco Germplasm Collection (Zan et al., 2025). Based on the prediction results, four accessions with low predicted total sugar content (TS1–TS4) and four accessions with high predicted total sugar content (TS5–TS8) were selected for field planting. The total sugar content of these eight accessions was then measured and compared with the predicted values. The Pearson correlation coefficient between the predicted and observed total sugar contents was 0.833, demonstrating the effectiveness of the GBM model for predicting total sugar content in tobacco leaves (Figure 6).

## Discussion

In this study, we conducted a comprehensive phenotypic and genetic analysis of total sugar content in tobacco and found substantial phenotypic variation among Chinese tobacco varieties, introduced materials, and local germplasm resources. Total sugar showed moderate heritability, and high-sugar genetic materials were identified within the germplasm collection, indicating considerable potential for improving total sugar content in major cultivated varieties such as Yunyan 87 and K326. Although GWAS identified one significant locus with a relatively large effect, total sugar in tobacco is still likely to be mainly controlled by many small-effect loci. The limited number of significant loci may be related to the small effects of individual loci and the conservative Bonferroni threshold, which can reduce the power to detect minor-effect variants for highly polygenic traits. By evaluating 16 genomic selection models in terms of prediction accuracy, computational resource use, and running time, we established a genomic selection framework for total sugar content, with the GBM model showing the best overall performance. The good performance of GBM may be related to its ability to capture complex and nonlinear relationships between SNP markers and phenotypic variation, which is suitable for traits with a polygenic genetic architecture.

Although this study provides a useful foundation for the genetic improvement of total sugar content in tobacco, several limitations remain. First, the field experiment was conducted in only one environment, and no multi-environment phenotypic correction was applied before model construction. Therefore, the current results mainly reflect model performance under the sampled environment. Second, although repeated five-fold cross-validation was performed, the cross-validation was based on random partitioning within the same germplasm population. Closely related accessions were not explicitly separated across folds, so the high prediction accuracy of GBM should be interpreted with caution. Third, the field validation included only eight accessions selected from the two extreme predicted groups, which may have increased the observed correlation between predicted and measured values. Therefore, this validation should be regarded as preliminary evidence rather than conclusive proof of model robustness. Future studies should use larger independent validation populations, multi-environment field trials, and population-structure- or kinship-based cross-validation to further evaluate the stability and practical value of the proposed model.

## Data Availability

The original contributions presented in the study are publicly available. This data can be found in the NCBI repository with the accession number PRJNA936601.
