# Supplementary material for: Construction and optimization of a genomic selection model for total sugar content in tobacco
Source: Front Genet. 2026 Jul 23;17:1886099. doi: 10.3389/fgene.2026.1886099 (PMC13446132; doi:10.3389/fgene.2026.1886099)
Supplement: Supplementary file 1 [file Table1.DOCX]

**Construction and optimization of a genomic selection model for total sugar content in tobacco**

Yue Yang^1^, Qiang Xu^1^, Jincun Fu^1^, Linjie Guo^2,4^, Zexiang Huang^6^, Huan Si^2^, Hao Wang^2^, Guanwang Shen^5,*^ Yanjun Zan^2,3,*^, Zongyu Hu^1,*^

1. China Tobacco Jiangsu Industrial Co., Ltd., Nanjing, 210000, China

2. Tobacco Research Institute, Chinese Academy of Agricultural Sciences, Qingdao, 266101, China

3. Department of Plant Physiology, Umeå Plant Science Center and Integrated Science Lab, Umeå University, Umeå, Sweden

4. College of Agronomy Qingdao Agricultural University, Qingdao, 266109, China

5. Integrative Science Center of Germplasm Creation in Western China (Chongqing), Science City, Biological Science Research Center, Southwest University, Chongqing 400716, China

6. Shandong Tobacco Corporation, Jinan 250101, Shandong Province

* Corresponding authors: Guanwang Shen, Integrative Science Center of Germplasm Creation in Western China (Chongqing), Science City, Biological Science Research Center, Southwest University, Chongqing 400716, China, Email: gwshen@swu.edu.cn; Yanjun Zan, Tobacco Research Institute, Chinese Academy of Agricultural Sciences, Qingdao, 266101, China, Email: [zanyanjun@caas.cn](mailto:zanyanjun@caas.cn); Zongyu Hu, China Tobacco Jiangsu Industrial Co., Ltd., Nanjing, 210000, China. Email: huzy707@sina.com

**Supplementary Table 1**. Best hyperparameter values of the genomic selection models for total sugar content in tobacco selected by grid search.

| **Model** | **Software** | **Parameters** | **Parameter range** |
| --- | --- | --- | --- |
| RR | Sklearn | default |  |
| LASSO | Sklearn | default |  |
| EN | Sklearn | default |  |
| GBLUP | rrBLUP | default |  |
| rrBLUP | rrBLUP | default |  |
| RKHS | BGLR | default |  |
| MKRKHS | BGLR | default |  |
| BA | BGLR | default |  |
| BB | BGLR | default |  |
| BC | BGLR | default |  |
| BRR | BGLR | default |  |
| BL | BGLR | default |  |
| GBM | Sklearn | learning_rate | 0.1, 0.01, 0.001 |
|  |  | n_estimators | 10, 50, 100, 200 |
|  |  | num_leaves | 2, 5, 20, 30, 50, 60 |
| RF | Sklearn | n_estimators | 100, 200 |
|  |  | max_features | sqrt, 0.5 |
| SVM | Sklearn | gamma | 'scale', 'auto' |
|  |  | C | 0.01, 0.1, 1, 5, 10 |
| DNNGP | Pytorch | batch_size | 16, 32, 64, 128 |
|  |  | learning_rate | 0.01, 0.001, 0.0001 |
